# Thalidomide Improves the Intestinal Mucosal Injury and Suppresses Mesenteric Angiogenesis and Vasodilatation by Down-Regulating Inflammasomes-Related Cascades in Cirrhotic Rats

**DOI:** 10.1371/journal.pone.0147212

**Published:** 2016-01-28

**Authors:** Tzu-Hao Li, Chia-Chang Huang, Ying-Ying Yang, Kuei-Chuan Lee, Shie-Liang Hsieh, Yun-Cheng Hsieh, Lin Alan, Han-Chieh Lin, Shou-Dong Lee, Chang-Youh Tsai

**Affiliations:** 1 Division of Allergy, Immunology, and Rheumatology, Department of Medicine, Taipei Veterans General Hospital, Taipei, Taiwan; 2 Department of Medicine, National Yang-Ming University School of Medicine, Taipei, Taiwan; 3 Division of General Medicine, Department of Medicine, Taipei Veterans General Hospital, Taipei, Taiwan; 4 Institute of Clinical Medicine, National Yang-Ming University School of Medicine, Taipei, Taiwan; 5 Division of Gastroenterology, Department of Medicine, Taipei Veterans General Hospital, Taipei, Taiwan; 6 Genomics Research Center, Academia Sinica, Taipei, Taiwan; 7 Cheng Hsin General Hospital, Taipei, Taiwan; Center for Cancer Research, National Cancer Institute, UNITED STATES

## Abstract

**Background and Aims:**

By blocking TNFα-related effects, thalidomide not only inhibits hepatic fibrogenesis but improves peripheral vasodilatation and portal hypertension in cirrhotic rats. Nonetheless, the investigation of thalidomide's effects on splanchnic and collateral microcirculation has been limited. Our study explored the roles of intestinal and mesenteric TNFα along with inflammasome-related pathway in relation to cirrhosis and the splanchnic/collateral microcirculation.

**Methods:**

Using *in vivo* and *in vitro* approaches, mechanisms of the effects of thalidomide on intestinal and mesenteric inflammatory, vasodilatory and angiogenic cascades-related abnormalities were explored in cirrhotic rats that had received 1-month thalidomide (C-T) treatment.

**Results:**

In cirrhotic rats, high tumor necrosis factor (TNF)α, vascular endothelial growth factor (VEGF) and nitric oxide (NO)x levels were associated with the NOD-like receptors protein 3 (NLRP3), IL-1β and caspase-1 inflammasome over-expression in splenorenal shunt and mesenteric tissues. The thalidomide-related inhibition of mesenteric and splenorenal shunt inflammasome expression was accompanied by a significantly decreased intestinal mucosal injury and inflammasome immunohistochemical staining expression. Suppression of various angiogenic cascades, namely VEGF-NOS-NO, was paralleled by a decrease in mesenteric angiogenesis as detected by CD31 immunofluorescence staining and by reduced portosystemic shunting (PSS) in C-T rats. The down-regulation of the mesenteric and collateral vasodilatory VEGF-NOS-NO cascades resulted in a correction of vasoconstrictive hypo-responsiveness and in an attenuation of vasodilatory hyper-responsiveness when analyzed by *in situ* perfusion of the superior mesenteric arterial (SMA) and portosystemic collaterals. There was also a decrease in SMA blood flow and an increase in SMA resistance in the C-T rats. Additionally, acute incubation with thalidomide abolished TNFα-augmented VEGF-mediated migration of and tube formation of human umbilical vein endothelial cells, which was accompanied by corresponding changes in inflammatory and angiogenic substances release.

**Conclusions:**

The suppression of inflammasome over-expression by chronic thalidomide treatment ameliorates inflammatory, angiogenic and vasodilatory cascades-related pathogenic changes in the splanchnic and collateral microcirculation of cirrhotic rats. Thalidomide seems to be a promising agent that might bring about beneficial changes to the disarrangements of peripheral, hepatic, splanchnic and collateral systems in cirrhosis.

## Introduction

Up-regulated vascular endothelial growth factor (VEGF) and nitric oxide (NO)-mediated splanchnic angiogenesis and vasodilatation, together with an attempt to divert the stagnant portal blood flow into the systemic circulation, are important for the development of hyperdynamic syndrome in cirrhotic portal hypertensive (PH) rats [1–7].

When there is a chronic inflammatory disease like cirrhosis present, TNFα brings about the stimulation of VEGF and NO-derived angiogenesis in order to supply nutrients and oxygen to the affected tissue [5,8]. The angiogenesis-related increase in endothelial surface area then creates an enormous capacity for interaction between inflammatory cytokines. Inflammasomes are large multiprotein complexes that are able to sense outside danger signals [9]. TNFα mediates the activation of the inflammasome and contributes to the progression of chronic inflammation [10–12]. TNFα and IL-1β are induced by similar stimuli in parallel and also share common intracellular signaling pathways [13]. NLRP3 specially regulates caspase-1 activation, which in turn allows the release of IL-1β to amplify the inflammatory response [14]. IL-1β is thought to function directly and indirectly to increase VEGF expression [15]. Via an IL-1β independent mechanism, the NOD-like receptors proteins (NLRPs) inflammasome also contributes to VEGF-mediated angiogenesis [16,17].

Thalidomide, a glutamic acid derivative with anti-angiogenic, anti-inflammatory and immunomodulatory properties, is able to suppress the production of TNF-α/IL-1β by activated immune cells [18]. To date, the outcome of treating pathogenic diseases involving TNF-α with thalidomide has resulted in very positive results [19].

Administration of thalidomide (50mg/kg/day) for two weeks beginning at two days before surgery in portal vein ligated rats has been found to ameliorate PH and peripheral vasodilatation, by means of an inhibition of VEGF-NO signaling [20]. An i*n vitro* study in BDL-cirrhotic rats showed that treatment with thalidomide (50mg/kg/day) for seven days enhanced portosystemic collaterals responsiveness to vasopressin [21]. Without hemodynamic measurement, administration of thalidomide (100mg/kg/day) for eight weeks resulted in an inhibition of the progression of cirrhosis in carbon tetrachloride-induced rats [22]. Additionally, administration of thalidomide (250mg/kg/day) for one month was found to decrease intrahepatic resistance in BDL-cirrhotic PH rats; however, this study did not investigate splanchnic and systemic hemodynamic changes [23,24]. Notably, the therapeutic effects of thalidomide have not yet been fully explored in relation to the splanchnic and collateral microcirculatory changes that occur with cirrhosis especially angiogenesis and hemodynamic disarrangement.

Accordingly, the current study investigates the potential effects of thalidomide on the TNFα-inflammasome-IL1β cascades-mediated effects associated with splanchnic and collateral abnormalities of cirrhotic PH rats together with an exploration of the mechanisms involved in these effects.

## Materials and Methods

The detail description was shown in **S1 Materials and Methods.**

### Duration of thalidomide administration

Thalidomide (200 mg/kg/day, oral gavage) or vehicle (distilled water, DW) were administered from the first day after common bile duct ligation (BDL) or sham operation for 1 month and the following experiments were performed as previously described [23,24]. All animals were purchased from Charles River Japan, Inc. (Yokohama, Japan) and received humane care in accordance with *Guide for the Care and Use of Laboratory Animals* (published by National Institute of Health). Two to three rats were raised in a 23cm height cage. In addition to freely access to food and water, rats were provided with small block of wood drilled with hold for chewing and with nesting materials for resting. Rats were euthanasia with 2–3 times the anesthetic dose. The experiment had been approved by the animal ethical committee of Taipei Veteran Hospital (IACUC number: 1020222, approved on 22/Feb/2013).

### Grouping

Four series of experiments were performed on BDL-cirrhotic rats that had randomly received vehicle (distilled water, C-V) and thalidomide for 4-week after BDL (C-T) (n = 6 in each group). In the first series, body weight (BW), mean arterial pressure (MAP), portal venous pressure (PVP), superior mesenteric arterial (SMA) flow and SMA resistance were measured. Meanwhile, *in situ* perfusion of SMA for measurement of concentration-response curve to arginine vasopressin (AVP) and acetylcholine was performed. The liver samples were collected for measuring levels of various pathogenic factors. In the second series, portosystemic shunting (PSS) was evaluated by the color microsphere method. Moreover, the mesenteric tissue was collected to measure various pathogenic factors. Simultaneously, terminal ileum was collected for H-E staining for the degree of mucosal damage by using a semi-quantitative grading system [25] and corresponding caspase-1/IL-1β immunohistochemical (IHC) staining. After blood withdrawing for various measurements, the mesenteric window vascular length and area with CD31 immunofluorescence (IF) staining were performed in the third series. Besides, splenorenal shunts of C-V and C-T rats were collected for various *mRNA*s and proteins measurement in the third series. In the fourth series, *in situ* perfusion of portosystemic collateral vessels in order to measure concentration-response curve to AVP and acetylcholine was performed. Also, the mesenteric tissue was collected to measure various *mRNAs*/proteins levels. Moreover, the acute effects of thalidomide on the migration of human umbilical vein endothelial cell cultures (HUVECs) and tube formation by HUVECs were measured. HUVECs were purchased from the Food Industry Research and Development Institute (Hsinchu, Taiwan).

### Effects of chronic thalidomide treatment on sham rats

Sham rats were randomly allocated to receive vehicle (S-V) or thalidomide (S-T) after sham operation. Non-cirrhotic (i.e., sham) rats do not develop PPS and, furthermore, our recent study reported that 1-month thalidomide treatment did not change the PVP and IHR [23]; therefore only MAP, SMA flow/resistance and mesenteric vascular length/area were evaluated to survey whether thalidomide treatment affected the relatively normal splanchnic circulation of our sham rats. Meanwhile, left renal vein tissues were collected from sham rats for various *mRNA*s and proteins measurement. Additionally, serum and hepatic tissues were collected for TNFα, IL-1β, caspase-1 and VEGF levels.

### Various hemodynamics and severity of intestinal mucosal injury and caspase-1/IL-1β expression

MAP, PVP, SMA blood flow/resistance and PSS were evaluated as previously mentioned [4,7,20,21]. The degree of mucosal damage of the terminal ileum lumen was assessed by semi-quantitative grading system [25]. Additionally, the IHC caspase-1/IL-1β staining indices of the ileal tissues were calculated as the product of the staining intensity score (0, 1, 2, and 3) and the proportion of positive cells (0, 1, 2, and 3). The degree of mucosal damage of the rat terminal ileum lumen and IHC caspase-1/IL-1β staining indices of the rat ileal tissues were scored in a blinded manner by one of the authors and by an expert.

### *In situ* microvessels perfusion

In both SMA and PS collaterals preparation, cumulative dose response curves of perfusion pressure change to AVP (10^−10^, 10^−9^, 10^−8^, 10^−7^, 10^−6^ M) and acetylcholine (ACh, 10^−12^ to 10^-7^M, endothelial dependent vasodilators) with phenylephrine (PEP, 10μM) pre-contraction were measured as previously reported [21].

### The *in vivo* evaluation of the mesenteric vascular density with IF study

The mesenteric angiogenesis (mesenteric window vascular length and area) were measured with flow probes and FITC-labeled CD31 IF staining [26].

### Various substances measurements

Serum, hepatic and mesenteric VEGF, TNFα, IL-1β, caspase-1, tetrahydrobiopterin (BH4) and total nitric oxide (nitrite+nitrate) levels were determined by commercially available ELISA kits (R&D system, Minneapolis, MN; BMS250, Bender MedSystems GmbH Vienna, Austria; Biovision; eBioscience) and colorimetric assay kit (Arbor assay kit, MI, USA).

### Various protein/*mRNA* expressions

Mesenteric tissue, left adrenal vein of sham rats and splenorenal shunts of BDL rats were collected for measurement of NLRP3, caspase-1, IL1β, VEGF, VEGFR2, phosphorylated (phos)-VEGFR-2, β-actin, TNFα, iNOS and eNOS expression using appropriate antibodies and primers (Table 1) purchased from Santa Cruz, Biotechnology, Inc.; R&D system, Minneapolis, MN; Abcam, Cambridge, MA, UK.

**Table 1:** Primer of rat gene used for quantitative realtime PCR analysis.

Gene name	Primers sequences
TNFα	For:5′-gctcacaatgtctgtgcttagag-3′; Rev:5′-gcagtagccacagctccag-3′
IL-1β	For:5′-tgtgatgaaagacggcacac-3′; Rev:5′-cttcttctttgggtattgtttgg-3′
NLRP3	For:5′-cagcgatcaacaggcgagac-3′;Rev:5′-agagatatcccagcaaacctatcca-3′
VEGF	For: 5′-ctacctccaccatgccaagt-3′; Rev:5′-gcagtagctgcgctgataga-3′.
CD31	For:5′-cttcaccatccagaaggaagagac-3′; Rev:5′-cactggtattccatgtctctggtg-3′
Angiopoetin-1	For: 5′-gggaggttggactgtaat-3′; Rev:5′-ctttatcccattcagttt-3′.
iNOS	For: 5′-agcatcacccctgtgttccaccc-3′; Rev:5′-tggggcagtctccattgcca-3′.
eNOS	For: 5′-ctgctgcccgagatatcttc-3′; Rev:5′-cag gta ctg cag tcc ctcct-3′
18S	For:5′-gtaacccgttgaaccccatt-3′; Rev:5′-ccatccaatcggtagtagcg-3

### HUVECs matrigel tube formation angiogenesis assay

The degree (angiogenic index) of *in vitro* formation of capillary like tube structure of HUVECs with buffer, VEGF (50ng/mL), VEGF+TNFα (0.05ng/mL) and VEGF+TNFα+thalidomide (10^-3^M) pretreatment for 36-hour were calculated among groups.

### Transwell chemotaxis filter assay for HUVECs migration

In order to assess the acute effects of thalidomide on the TNFα-augmented VEGF-mediated HUVECs migration [migration index (MI)], thalidomide (10^−3^ M) and TNFα (0.05ng/mL) were co-incubated with VEGF (50ng/mL), and the results were compared to the vehicle-co-incubated group.

### Various measurements in the HUVEC system

The protein lysates, supernatant and total *RNA*s were obtained from cultured HUVECs (5×10^6^) with buffer, VEGF, VEGF+TNFα and VEGF+TNFα+thalidomide pre-treatments. Then, various proteins (NLRP3, caspase-1, IL-1β, VEGFR2 and phos-VEGFR2), total nitric oxide (NOx), angiopoietin-1, IL-1β, and caspase-1 levels, *mRNA* expressions of CD31, angiopoietin-1, iNOS and eNOS were measured with appropriate primers listed in Table 1.

### Statistical analysis

Results were expressed as mean±SEM. Statistical analyses were perfomred by means of unpaired Student’s *t* test or one-way ANOVA, as appropriate. Results were considered statistically significant at a two-tailed *P* value less than 0.05.

## Results

### Chronic hemodynamic effects of thalidomide

Table 2 showed that the BW, MAP and HR were not modified in BDL-cirrhotic (C-T) and sham (S-T) rats that received chronic thalidomide treatment compared to those that had received vehicle. Nonetheless, the PVP and SMA flow of C-T rats compared to C-V rats were significantly reduced. Significantly, the SMA resistance was increased in C-T rats compared to that in C-V rats. There were also significant decreases in plasma, hepatic and mesenteric levels of TNFα, indicating that the current dosage of thalidomide used to treat the cirrhotic rats were appropriate (Table 3).

**Table 2:** Hemodynamics in BDL rats with thalidomide treatment.

	BDL-cirrhotic rats(n = 6)		Sham rats(n = 5)	
	Vehicle (C-V)	Thalidomide (C-T)	Vehicle (S-V)	Thalidomide (S-T)
Body weight (g)	385±8	390±10	410±9.7	400±13
Mean arterial pressure (MAP, mmHg)	96.8±5.7	101.2±4.6	118±9	109±12
Heart rate (beats/min)	341±16	329±17	238±19	227±28
Superior mesenteric arterial blood flow (SMA flow, mL/min)	11±1.7	8.2±0.9*	5.4±0.8	5.6±1.4
Superior mesenteric arterial resistance (SMA resistance, mmHg/mL/min/100g)	7.4±2	10.8±1.3^#^	-	-
Portal venous pressure (PVP, mmHg)	16±1.9	12.5±2.2^#^	-	-
Portal systemic shunting (PSS, %)	80±14	67.2±5*	-	-

*P<0.05

^#^*P*<0.001

because non-cirrhotic sham rats do not develop PPS and our recent study reported that 1-month thalidomide did not change the PVP, only MAP, SMA flow were evaluated to survey whether thalidomide affected the relatively normal splanchnic circulation. SMA resistance is calculated from the formula of (MAP-PVP)/SMA flow. So, the SMA resistance of sham rats cannot be calculated without PVP data.

**Table 3:** Plasma, Hepatic and Mesenteric Levels of Various Pathogenic Factors.

	BDL-cirrhotic rats(n = 6)		BDL-cirrhotic rats(n = 6)		BDL-cirrhotic rats(n = 6)		sham rats(n = 6)		sham rats(n = 6)		sham rats(n = 6)	
	Vehicle(C-V)	Thalidomide(C-T)	Vehicle(C-V)	Thalidomide(C-T)	Vehicle(C-V)	Thalidomide(C-T)	Vehicle(S-V)	Thalidomide(S-T)	Vehicle(S-V)	Thalidomide(S-T)	Vehicle(S-V)	Thalidomide(S-T)
	Serum (pg/mL)		Hepatic tissue (pg/g)		Mesenteric tissue (pg/g)		Serum (pg/mL)		Hepatic tissue (pg/g)		Mesenteric tissue (pg/g)	
TNFα	21.3±2.9^**δ**^	15.4±1.8	385±17^**δ**^	264±11	306±22^**δ**^	241±19	10.5±0.9	9.4±1.8	213± 6.8	198.2 ±10.3	201.2± 8.8	193.5±9.8
IL-1β	42.1±3.8	37.5±7.4	523±46^**δ**^	318±9	497±35^**δ**^	327±58	23.5±1.7	22.1±3.1	302.5±18.4	289.2 ±2.7	195.1±10.6	186.1±17.4
Caspase-1	168.4±22.3	157.3±29.5	311±32^**δ**^	196±16	242±16^**δ**^	131±17	83.5±7.3	79.8±10.6	168.6±5.4	151.6±12.8	105.3±7.5	99.3±12.3
VEGF	16.8±3.1	15.4±2.1	172±8^**δ**^	124±7	256±14^**δ**^	174±23	9.3±0.9	8.2±1.1	102.3±3.2	99.6±8.1	123.1±4.2	114.1±18.2

^δ^ *P* < 0.05 *vs*. C-T rats

### Plasma, hepatic and mesenteric levels of various substances

Overall, higher serum, hepatic and mesenteric TNFα, IL-1β, caspase-1 and VEGF levels were noted in the C-V rats compared to the S-V rats (Table 3). Chronic thalidomide treatment of C-T rats resulted in a simultaneous inhibition of hepatic and mesenteric TNFα levels, together with a suppression of inflammasomes, specifically the levels of IL-1β and caspase-1 (Table 3). Notably, hepatic and mesenteric VEGF levels were significantly reduced by chronic thalidomide treatment in the C-T rats compared to the C-V rats. Significantly, the higher *mRNA*/protein expressions of TNFα and eNOS were accompanied by the higher BH4 (179.4±25 *vs*. 118.6±21 pmol/g protein, *P*<0.05) and NOx (8.13±0.09 *vs*. 4.11±0.32μmol/mg, *P*<0.01) levels in C-V rats than those in S-V rats. Furthermore, the down-regulation of eNOS by chronic administration of TNFα blocking agent thalidomide was associated with the suppression of mesenteric BH4 (148.5±13 *vs*. 179.4± 23 pmol/g, *P*<0.05) and NOx (6.21±0.3 *vs*. 8.13±0.09 μmol/mg, *P*<0.05) levels in C-T rats compared to those of C-V rats. Nonetheless, the BH4 (118.6±21 *vs*. 102.4±33 pmol/g) and NOx (4.11±0.32 *vs*. 3.98±0.78 μmol/mg) levels in mesenteric tissue were not different between S-V and S-T rats. The suppression of serum, hepatic and mesenteric TNFα levels was found not to be associated with decreases in serum, hepatic and mesenteric levels of IL-1β, caspase-1, VEGF, BH4 and NOx in the S-T rats.

### Effects of chronic thalidomide treatment on *in situ* SMA and portosystemic collaterals perfusion

**Fig 1:**
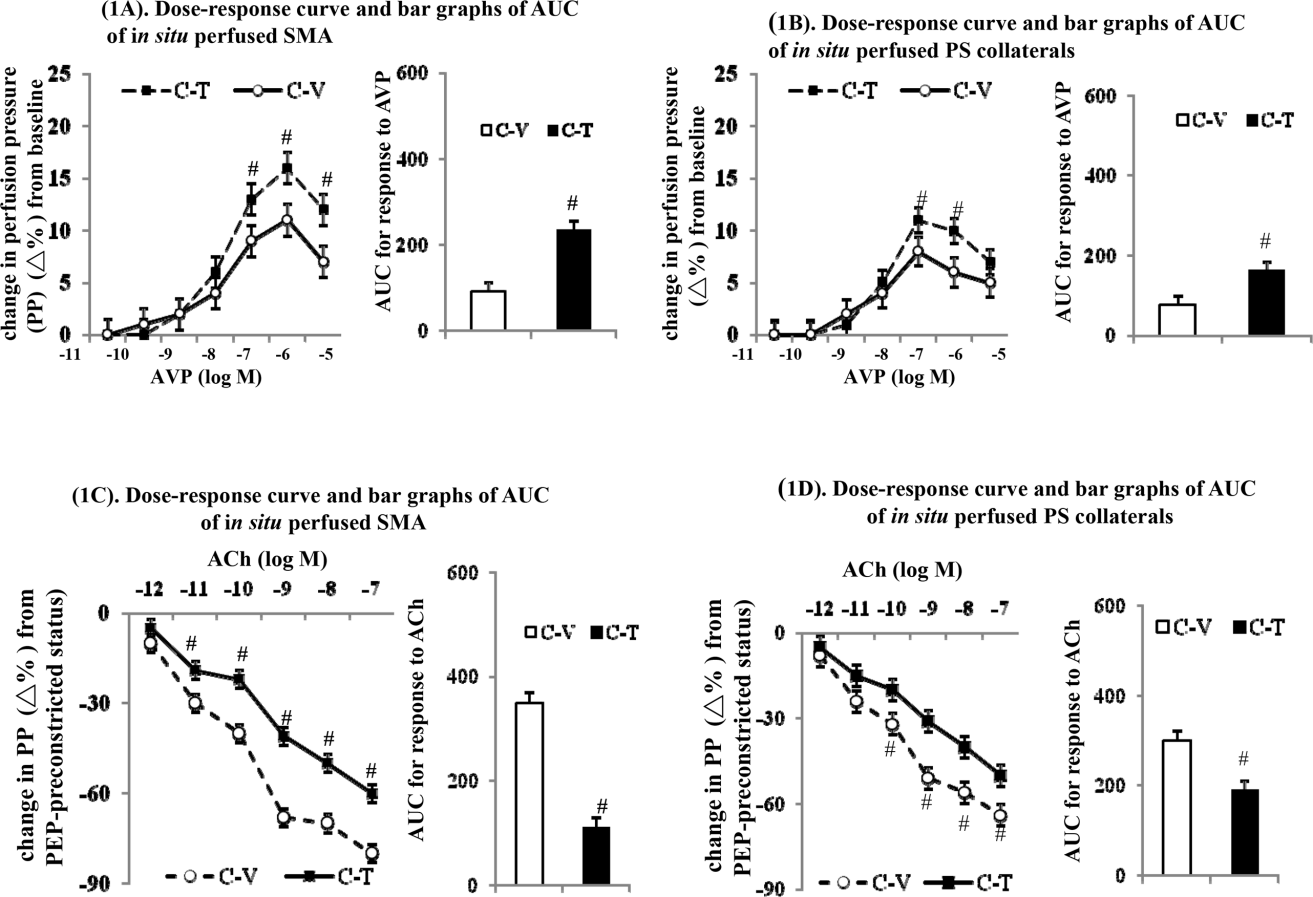
Dose-response curves and area under curves (AUC) of *in situ* perfusion of cirrhotic microvessels. Chronic thalidomide treatment improves vasoconstrictive hypo-responsiveness to arginine vasopressin (AVP) (**A**,**B**) and attenuates vasodilatory hyper-responsiveness to acetycholine (ACh) (**C**,**D**) in superior mesenteric arteries (SMA) and portosystemic collateral vessels (PS collaterals); ^#^: *P* < 0.05 *vs*. C-V group.

In the C-V rats, a significant vasodilatory state could be noted with lower basal vascular tone when i*n situ* SMA perfusion (baseline perfuson pressure, 17.5±2.3 *vs*. 21.6±1.4mmHg, *p* < 0.001) and portosystemic collaterals perfusion (16.1±2.4 *vs*. 23.8±1.5mmHg, *p* < 0.001) were carried out compared to those in C-T rats. Compared to the vehicle-treated cirrhotic (C-V) rats, the improved vasoconstrictor hypo-responsiveness [a higher vasoconstritive response curve and a greater area under curve (AUC) in response to AVP] were observed when SMA perfusion of C-T rats was carried out (Fig 1A). Correspondingly, there was also an amelioration of the vasodilatory hyper-responsiveness [a lower relaxation response curve and smaller AUC in response to acetylcholine] when SMA perfusion of C-T rats relative to C-V rats was investigated (Fig 1C).

Similarly, the correction of vasoconstrictor hypo-responsiveness to AVP was accompanied by an amelioration of the vasodilatory hyper-responsiveness to acetylcholine in portosystemic collaterals perfusion of C-T rats compared to C-V rats (Fig 1B and 1D). Among the C-T rats, the magnitude of the restoration of the vasoconstrictive responsiveness to AVP was higher with SMA perfusion than with the portosystemic collaterals perfusion (155±3% *vs*. 108±4%, *p*<0.01). Correspondingly, the degree of the attenuation of the vasodilatory hyper-responsiveness to acetylcholine was higher during SMA perfusion of C-T rats compared to portosystemic collaterals perfusion of C-T rats (81±2% *vs*. 37±5%, *p*<0.05).

### PSS and mesenteric angiogenesis

**Fig 2:**
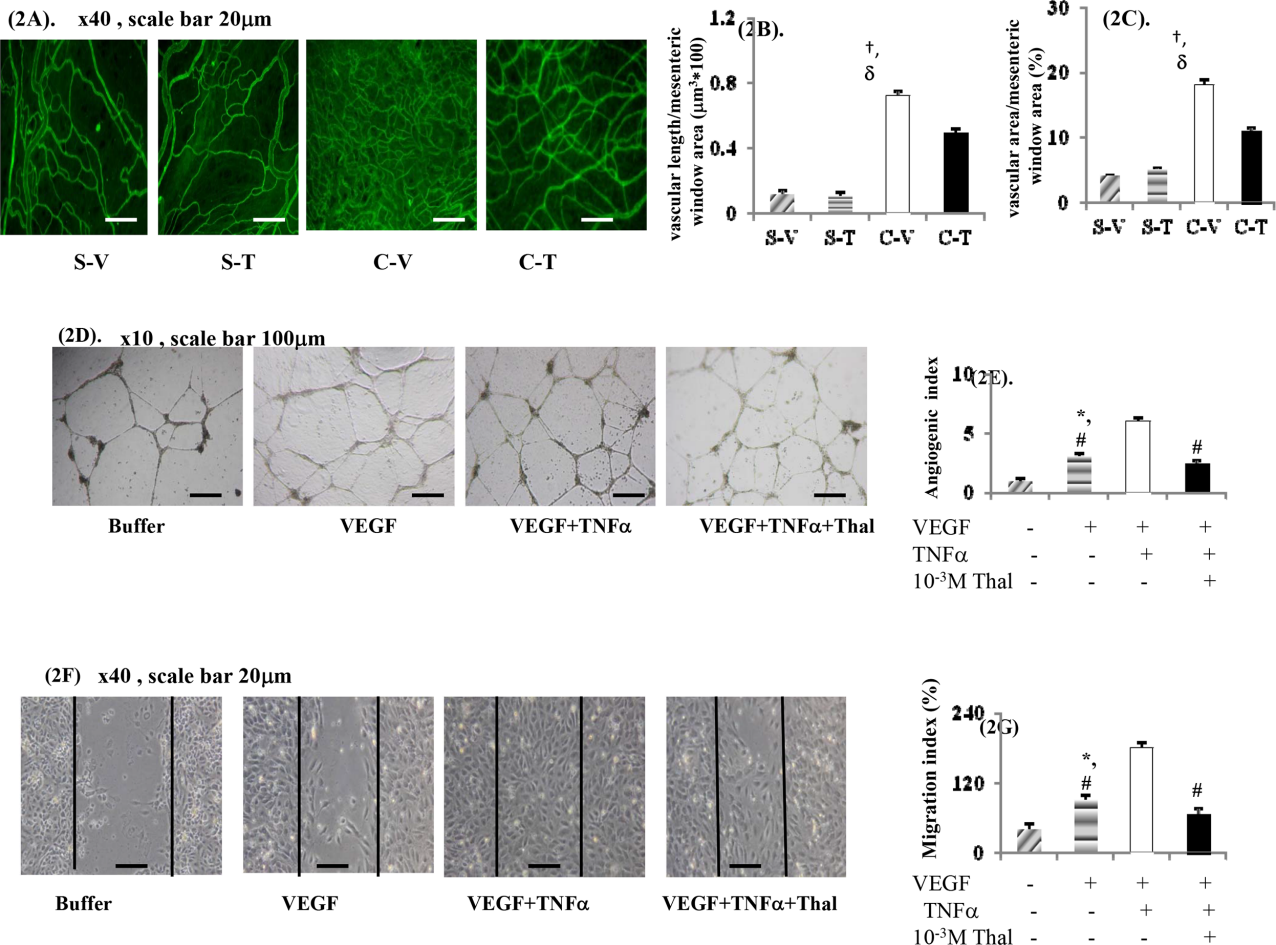
*In vivo* and *in vitro* effects of thalidomide treatment on mesenteric and human umbilical vein endothelial cells (HUVECs)-induced angiogenesis. Representative CD31 immunofluoresence (IF) staining angiogenesis images (**A**) and bar graphs of vascular length (**B**) and area (**C**) in mesenteric window. Representative images and bar graph of HUVECs tube formation (**D,E**) and migration (**F,G**) assays after 36hr of treatment; ^†^: *P*<0.05 *vs*. S-V; ^δ^: *p*< 0.05 *vs*. C-T rats;^#^: *p* < 0.01 *vs*. VEGF+TNFα; *: *p* < 0.05 *vs*. buffer group.

When C-T rats were compared to C-V rats, Table 2 shows that the severity of PSS was significantly alleviated by chronic thalidomide treatment in the former group. Compared with C-V rats, chronic thalidomide treatment significantly decreased the vascular length and area per unit field of the mesenteric window when CD31 IF-stained images of C-T rats were studied (Fig 2A–2C). Nonetheless, the mesenteric vascular density was found not to be different between the S-V and S-T rats.

Morphologically, compared with the C-V rats, there was a significant decrease in the numbers of vessels that showed an irregular and loose distribution in the C-T rats. In other words, less tortuousity and a low numbers of vessels were noted in the C-T rats (Fig 2A). However, the general morphology and intensity of small vessels within the mesenteric window were not different between the S-V and S-T rats.

### *mRNA*/protein expressions levels in the left adrenal veins of sham rats and in the splenorenal shunts of BDL-cirrhotic rats

**Fig 3:**
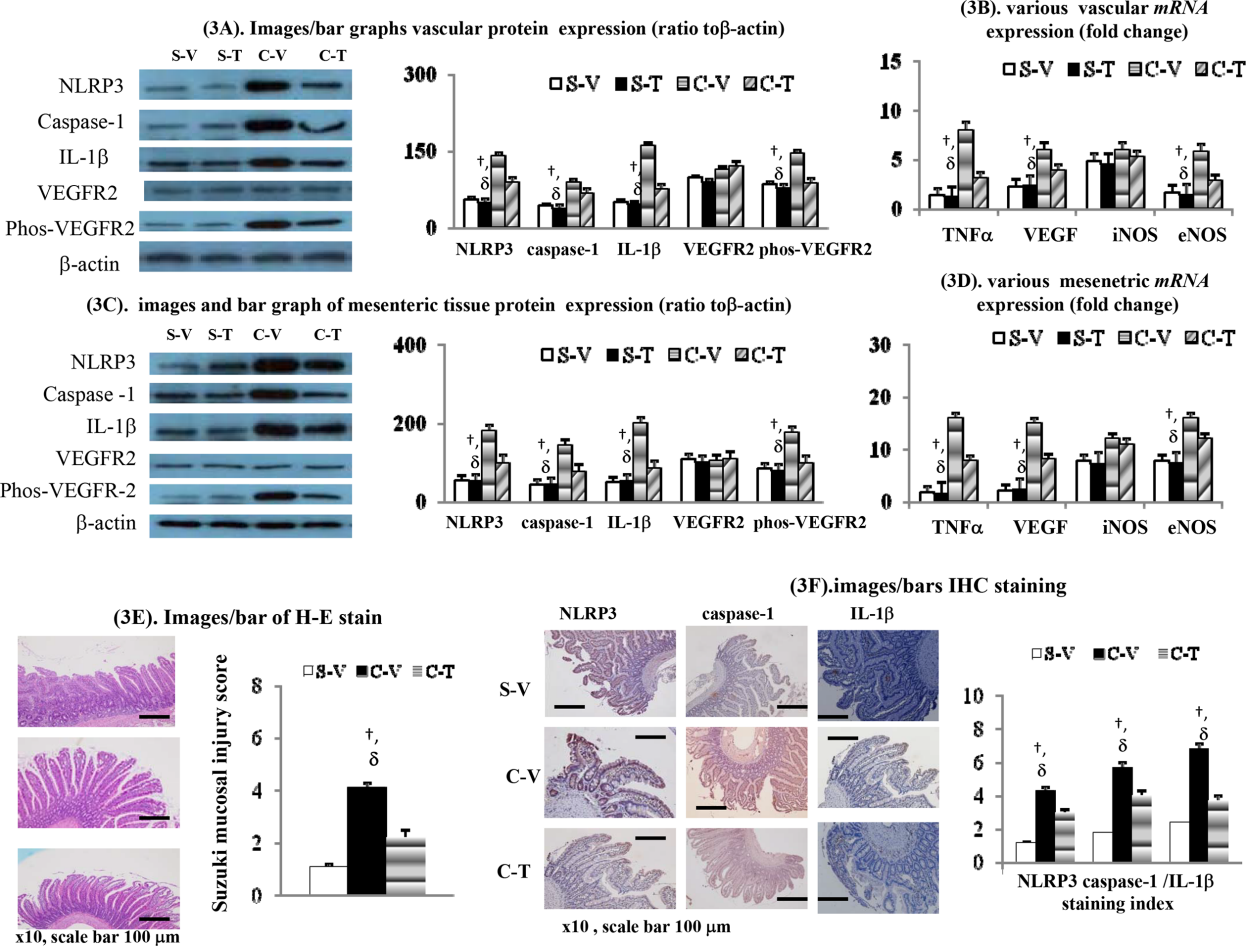
Effects of thalidomide treatment on various cirrhotic vascular/mesenteric/intestinal inflammatory, angiogenic and vasodilatory markers. Protein and *mRNA* expressions in splenorenal shunts (BDL) and left renal vein (sham). (**A**,**B**), and mesenteric tissues (**C**,**D**); (**E**).H-E staining for severity of mucosal injury (suzuki score) of small intestine; (**F**). IHC staining for inflammasome expression (NLRP3/caspase-1/IL-1β) of ileum. †*p*<0.05 *vs*. S-V rats; δ *p*< 0.05 *vs*. C-T rats. Genes were normalized to 18S RNA as an internal control.

Fig 3A and 3B reveals that vascular *mRNA* and protein expression levels of TNFα, various inflammasome (NLRP3, cascapse-1 and IL-1β) and a number of angiogenic markers (phos-VEGFR2, VEGF and eNOS) were significantly higher in the C-V rats (splenorenal shunts) compared to the S-V rats (left adrenal veins). Apart from reducing the vascular *mRNA* and protein expression levels of TNFα in C-T rats (splenorenal shunts), chronic thalidomide treatment also down-regulated the expressions levels of the aforementioned inflammasome *mRNAs* and proteins, together with the various angiogenic markers. Nonetheless, the vascular expression levels of iNOS *mRNA* and VEGFR2 protein were not different between C-V (splenorenal shunts), S-V (left adrenal veins), S-T (left adrenal veins) and C-T (splenorenal shunts) rats (Fig 3B).

### *mRNA* and protein expression in mesenteric tissue

Fig 3C and 3D reveals that *mRNA* and protein expression levels of TNFα, various inflammasome (NLRP3, cascapse-1 and IL-1β) and angiogenic markers (phos-VEGFR2, VEGF and eNOS) were significantly higher in the mesenteric tissue of C-V rats compared to S-V rats. In addition to reducing expression of TNFα in the mesenteric tissue of C-V rats, chronic thalidomide treatment also down-regulated expression of the various inflammasome *mRNAs* and proteins in parallel with a reduction in the angiogenic markers. However, there was no changes in the expression levels of iNOS *mRNA* and VEGFR2 protein when the mesenteric tissue of S-V, S-T, C-V and C-T rats were compared (Fig 3D).

### Intestinal mucosal injury and inflammasome IHC staining

Fig 3E showes that more severe intestinal mucosal injury (a higher Suzuki mucosal injury score) was accompanied by higher expression levels of various intestinal inflammasome, namely caspase-1, IL-1β and NLRP3, in C-V rats compared to S-V rats as assessed by the IHC staining indices for caspase-1,IL-1β and NLRP3 (Fig 3F). Chronic thalidomide treatment effectively suppressed intestinal mucosal injury and caspase-1/IL-1β/NLRP3 inflammasome over-expression in C-T rats compared to C-V rats.

### Direct *in vitro* effects of thalidomide on HUVECs

VEGF pre-incubation is able to induce migration of HUVECs and tube formation by HUVECs (Fig 2D–2G). Furthermore, the concomitant acute TNFα incubation significantly augmented the VEGF-induced migration of HUVECs and tube formation by HUVECs. Notably, the TNFα-augmentation of VEGF-mediated HUVECs migration and tube formation was abolished by silmutaneous thalidomide pre-incubation.

**Fig 4:**
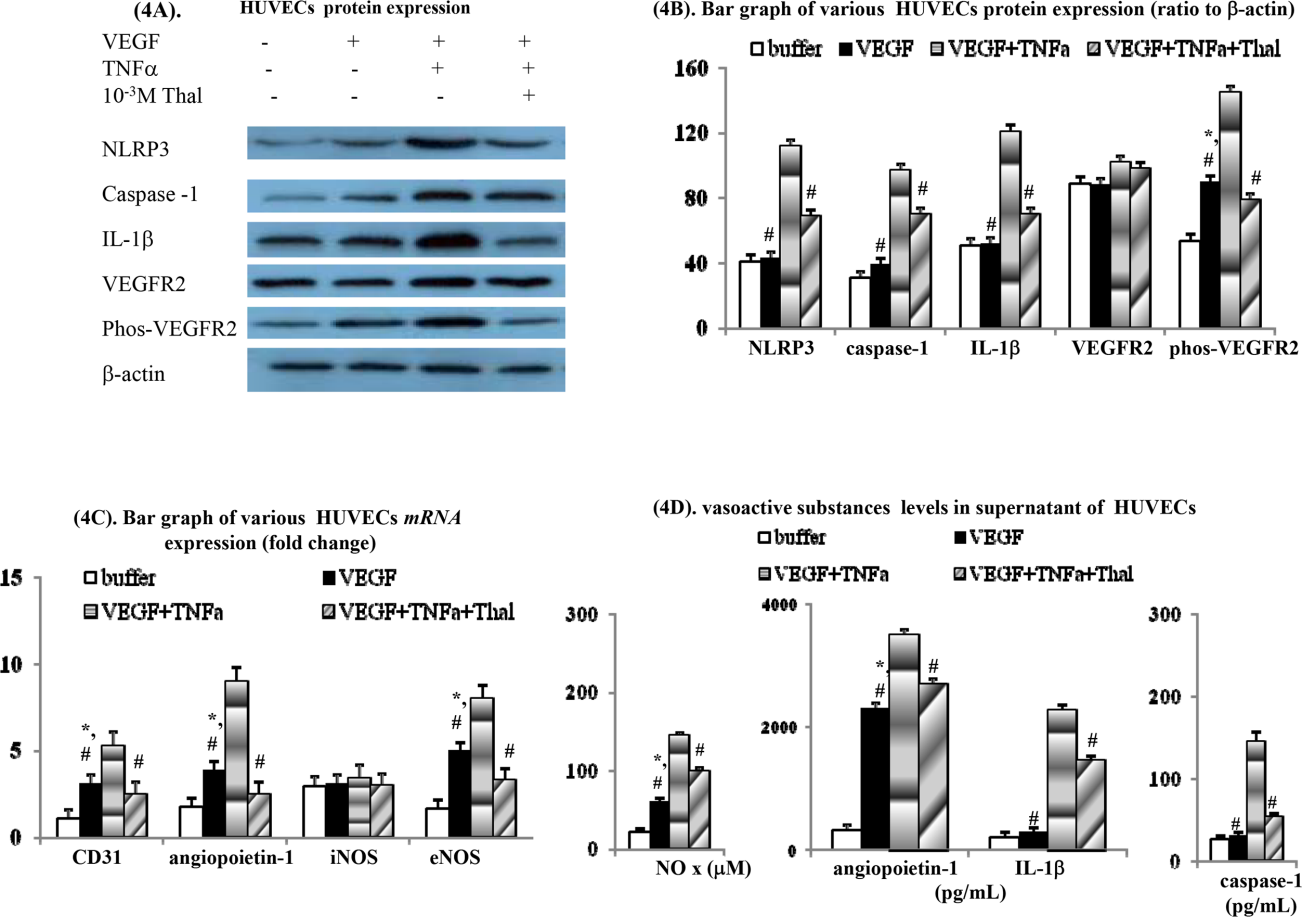
Acute effects of thalidomide on various cirrhotic vascular/mesenteric/intestinal inflammatory and angiogenic markers in human umbilical vein endothelial cells (HUVECs) system. Various protein (**A-B**), *mRNA* (**C**) and cytokines (**D**) levels in supernatant of HUVECs with different treatments. #: *p* < 0.01 *vs*. VEGF+TNFα; *: *p* < 0.05 *vs*. buffer group.

Within the HUVECs culture system, the *mRNA* and protein expression levels of phos-VEGFR2, CD31, angiopoietin-1 and eNOS were significantly up-regulated after acute VEGF pre-incubation. Furthermore, concomitant TNFα pre-incubation also augmented the aforementioned VEGF-upregulated *mRNAs* and proteins expression levels in the HUVECs (Fig 4A–4C). Compared to the buffer and VEGF only groups, the protein expression levels of NLRP3, caspase-1 and IL-1β were also significantly up-regulated during simultaneous TNFα pre-incubation of HUVECs. In parallel, the up-regulation of phos-VEGFR2, CD31, angiopoietin-1 and eNOS at the *mRNA* and protein expression levels by VEGF was found to be further increased by co-pre-incubation with TNFα.

Additionally, both TNFα-augmented NLRP3, caspase-1 and IL-1β, and VEGF/VEGF+TNFα-upregulated phos-VEGFR2, CD31, angiopoietin-1 and eNOS at the *mRNA* and protein expression levels were suppressed by concomitant acute thalidomide pre-incubation.

Acute VEGF pre-incubation also stimulated release of angiopoietin-1 and NOx into the supernatant of the cultured HUVECs (Fig 4D). Interestingly, simultaneous TNFα pre-incubation increased the release of VEGF-stimulated angiopoietin-1 and NOx into the supernatant of the cultured HUVECs (Fig 4D). In contrast, the release into the HUVECs supernatant of two inflammasomes, IL-1β and caspase-1, was not only induced by VEGF alone, but also stimulated by TNFα co-incubation. Overall, the release of angiopoetin-1, NOx, IL-1β and caspase-1 by VEGF+TNFα stimulation was significantly inhibited by concomitant pre-incubation with thalidomide in the HUVEC culture system.

## Discussion

Our study characterized by exploring both the *in vivo* and *in vitro* effects of chronic thalidomide treatment on the vasodilatation, angiogenesis and inflammation as they affect the splanchnic and collateral systems of cirrhotic rats.

In a manner that is different from previous reports [18,20,21,23], our study provides hemodynamic measurement data showing that the PSS and SMA blood flows and resistances were significantly decreased when the chronic TNFα blocking agent thalidomide was used to treat cirrhotic rats. It has been reported that TNFα is involved in the NO-mediated mesenteric vasoconstrictive hypo-responsiveness in cirrhotic rats [27,28]. In addition to reconfirming the enhancement of vasoconstrictor responsiveness of the portosystemic collaterals [21], our study also discovered that there was an attenutation of the vasodilatory hyper-responsiveness of the SMA and portosystemic collaterals when cirrhotic rats were treated with thalidomide. In parallel there was down-regulation of eNOS expression together with a decrease in local mesenteric NOx release when perfused SMA and portosystemic collaterals were examined in cirrhotic rats are treated with thalidomide. It has been previously documented that attenuation of peripheral vasodilatation [20], and amelioration of splanchnic vasodilatation, which are observed in our thalidomide-treated cirrhotic rats, bring about an improvement in portal hypertension.

In our current study, acute thalidomide incubation significantly diminished the TNFα-enhanced VEGF-mediated migration of HUVECs as well as tube formation by HUVECs. TNFα and thalidomide have been reported to inhibit the migration of endothelial cells and capillary tube formation by endothelial cells [29,30]. The angiogenic markers CD31 and angiopoietin-1 are constitutively expressed on endothelial cells [31,32]. Angiopoietin-1-induced angiogenesis has been found to be dependent on VEGF. In our study, the over-expression of CD31 on the cirrhotic rat mesenteric window was found to be significantly suppressed by thalidomide treatment. In the supernatant of our HUVECs culture system, the release of two angiogenic markers, CD31 and angiopoietin-1, stimulated by either TNFα alone or by VEGF+TNFα co-stimulation was decreased by acute thalidomide incubation.

TNFα can directly increase the activity of eNOS-NO by upregulating BH4 production [33]. In our study, the down-regulation of eNOS by chronic administration of TNFα blocking agent thalidomide was associated with the suppression of mesenteric BH4 and NOx levels in cirrhotic rats. Actually, it had been suggested that mesenteric vasculature BH4 levels were significantly positively correlated with TNFα levels in mesenteric lymph node (MLN) in cirrhotic rats [28]. Taken together our and previous studies, the down-regulation of eNOS-NO activity by chronic blocking with thalidomide might be mediated by simultaneous suppression of BH4 production in mesenteric tissue.

It seems likely that TNFα activates the NO synthase-NO signals that are involved in the migration of and angiogenesis by endothelial cells [34,35]. By blocking these TNFα–related effects, thalidomide is able to attenuate NO-mediated angiogenesis; this seems to occurs via the blocking of endothelial cells migration, which has been shown to occur in a dose-dependent manner [30]. In parallel, thalidomide has TNFα-independent anti-angiogenic properties that results in a direct suppression of the activity of the angiogenic NOS-NO signals [36]. In supernatant of our HUVECs, the VEGF-stimulated of TNFα-augmented eNOS expression and NOx release significantly inhibited by acute thalidomide incubation. Furthermore, this acute thalidomide incubation-related suppression of eNOS-NO signals was associated with the down-regulation of phos-VEGFR2, CD31 and angiopoietin-1 into the supernatant of the HUVECs. Furthermore, above changes were accompanied by a suppression of the migration of HUVECs and tube formation by HUVECs.

The release of inflammatory cytokines and inflammasomes has been shown to further stimulate angiogenesis and vasodilatation in various tissues [5,6,13,15,16]. Our study explored the effects of the anti-inflammatory agent thalidomide on the pathogenic changes in splanchnic microcirculation of cirrhotic rats in detail. Silmutaneously, in our cirrhotic rats, chronic thalidomide treatment inhibited the mesenteric inflammasome while reducing the release of various angiogenic and vasodilatory substances, including IL-1β, casapse-1, NLRP3, CD31, angiopoietin-1, VEGF, phos-VEFGR2 and NO. The thalidomide treatment-related suppression in cirrhotic rats of aforementioned increased inflammatory, angiogenic and vasodilatory factors on SMAs and portosystemic collaterals were accompanied by decreased SMA flow/portosytemic shunting (PSS), which was paralleled by an increase in SMA resistance. In other words, chronic thalidomide treatment decreased the elevation of these pathogenic factors and improved structural (angiogenesis) and functional (vasodilatory hyperresponsiveness) abnormalities present in cirrhotic PH rats.

During cirrhosis, persistent intestinal mucosal inflammatory injury results in a stimulation of TNFα production by gut-derived endotoxin activated inflammatory cells [37]. TNFα is able to cause apoptosis of intestinal epithelial cells and this exaggerates the severity of mucosal injury [38]. In NLRP3 inflammasome-mediated inflammation, TNFα and IL-1β stimulate caspase-1 activation [10,11]. The anti-inflammatory activity of thalidomide has been reported to be mediated via the inhibition of activated caspase-1 in mice [39]. Activation of caspase-1 had been reported to aggravate intestinal mucosal injury [40].

Inflammasomes are able to stimulate NOS to release NO [41], which would seem to exaggerate intestinal mucosal injury [42]. In our cirrhotic rat model, treatment with the TNFα blocking agent thalidomide resulted in the suppression of mesenteric tissue NOS-NO signaling, in parallel with an inhibition of intestinal mucosal injury. Based on our findings, the BDL-cirrhotic model revealed that over-expression of mesenteric/intestinal TNFα, IL1β and increased inflammasome expressions were accompanied by significant intestinal inflammation and increased mucosal injury. So, it is reasonable to conclude that the thalidomide treatment-related inhibition of mesenteric/intestinal TNFα, IL1β and inflammasome-related marker expression, based on analysis using serological, Western blotting and IHC analysis, are associated with the decrease in the severity of intestinal mucosal injury in our cirrhotic rats.

Thalidomide had been shown to have both anti-inflammation and anti-angiogenesis effects [22,28]. It has also been reported that the activation of caspase-1 links inflammation with angiogenesis [40]. Our *in vivo* study revealed that the chronic thalidomide-related caspase-1 inhibition together with the alleviation of intestinal mucosal injury in cirrhotic rats are accompanied by suppression of mesenteric angiogenesis, as measured by CD31-IF-staining and phos-VEGFR2 Western blot analysis. Our *in vitro* findings indicated that the acute thalidomide incubation-related suppression of TNFα-activated caspase-1 and cytokine expression are associated with decreases in the levels of phos-VEGFR2, CD31 and angiopoietin-1 in supernatant of treated HUVECs. Furthermore, the above changes were accompanied by a suppression of the VEGF-stimulated migration of HUVECs and a suppression of HUVECs tube formation. These findings suggest that there is a paracrine regulatory loop in HUVECs that involves inflammasomes, cytokines and angiogenic factors and that this is able to be suppressed by blocking TNFα expression using thalidomide.

## Conclusions

**Fig 5:**
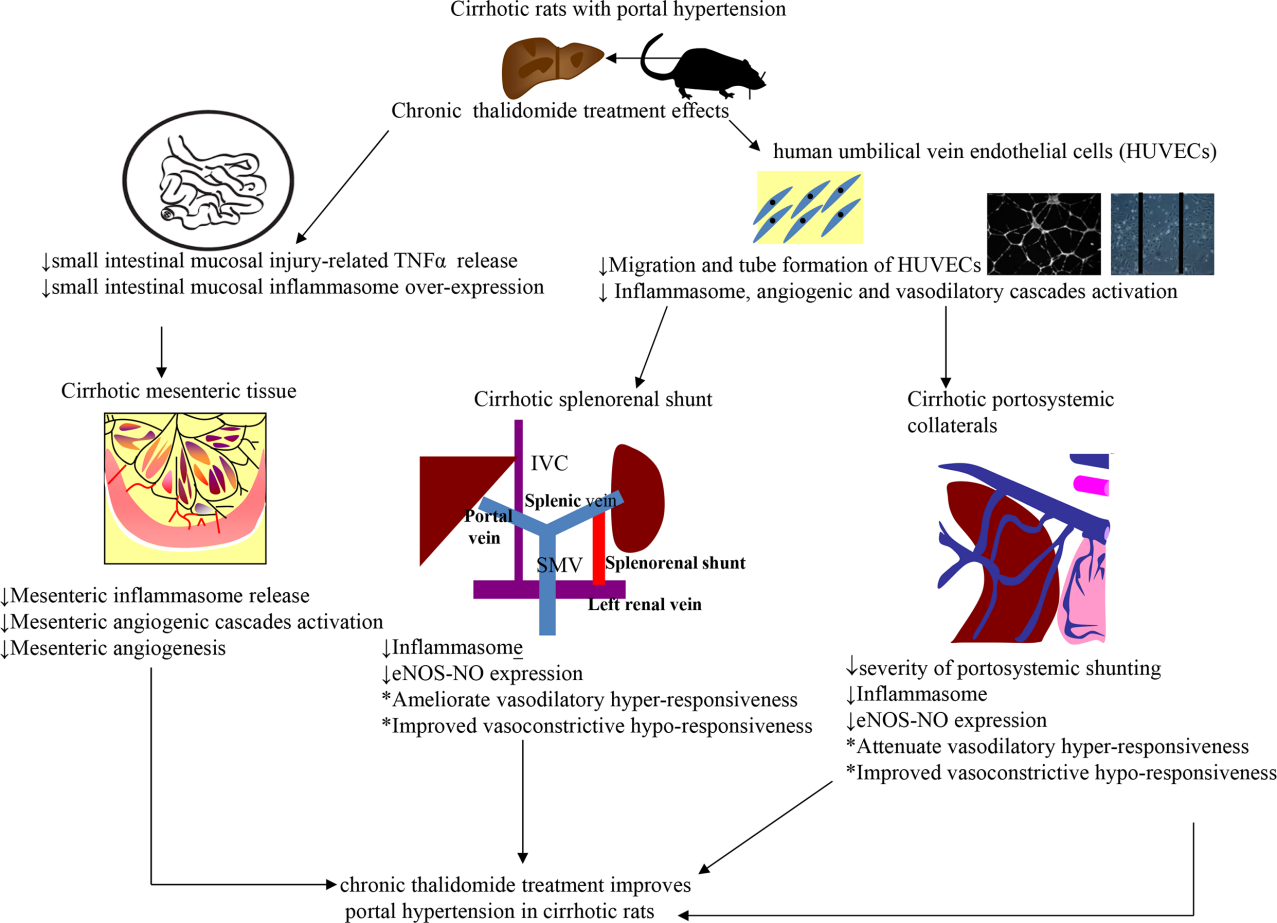
Schematic representative hypothesis of chronic thalidomide treatment effects on cirrhotic rats of our study. TNFα: tumor necrosis factor-α; IVC: inferior vena cava; SMV: superior mesenteric vein; eNOS: endothelial nitric oxide synthase; NO: nitric oxide.

Our study has shown that intestinal mucosal injury was accompanied by increased inflammasome expression and TNFα over-expression in the intestinal/mesenteric tissues and splanchnic/portosystemic collateral vessels of cirrhotic rats. Accordingly, our findings suggested that inflammasomes participate in the positive regulatory loop among intestinal inflammation, intestinal mucosal injury, mesenteric angiogenesis and splanchnic vasodilatation of cirrhosis (Fig 5). In addition to down-regulation of the TNFα-VEGF-NOS-NO pathway, thalidomide also brings about both the structural and functional beneficial effects that modify the splanchnic and portosystemic collaterals systems and these changes are contributed to by inflammasome suppression within the mesenteric and intestine tissues (Fig 5).

## Supporting Information

S1 Materials and Methods (DOCX)
