# Supplementary material for: Thalidomide Improves the Intestinal Mucosal Injury and Suppresses Mesenteric Angiogenesis and Vasodilatation by Down-Regulating Inflammasomes-Related Cascades in Cirrhotic Rats
Source: PLoS One. 2016 Jan 28;11(1):e0147212. doi: 10.1371/journal.pone.0147212 (PMC4731147; doi:10.1371/journal.pone.0147212)
Supplement: S1 Materials and Methods — (DOCX) [file pone.0147212.s001.docx]

**S1 Materials and Methods.**

**SMA blood flow and resistance**

The hemodynamic measurements were performed as previously. Under ketamine anesthesia (100mg/kg intramuscularly), the femoral artery was cannulated with PE-50 tube to monitor mean arterial pressure (MAP). The portal vein was cannulated via an ileal vein for measurement of PVP. The rectal temperature was maintained at 37℃ using a heating pad. All pressure were recorded using a multichannel recorder (Gould Inc., CA, USA) and connected to a flowmeter (Transonic In., NY, USA) for continuous monitoring of SMA blood flow. SMA resistance (mmHg/ml/min 100g BW) was calculated by (MAP-PVP)/SMA flow per 100g BW.

**Color microsphere method for PSS analysis**

As described previously, 300,000 yellow microspheres (15µm diameter, Triton Technologies, San Diego, CA, USA) were slowly injected into the spleen [1]. The rats were then euthanized and the livers and lungs dissected. The number of microspheres in each tissue was determined following the protocol provided by the manufacturer; 3,000 blue microspheres served as the internal control. Tissue was digested overnight with 1M KOH at 60℃ and thoroughly sonicated. After centrifugation, the supernatant was removed, and the pellets were washed once with 10% Triton X-100 and twice with acidified ethanol. After that, pellets containing the microspheres were allowed to dry overnight. The microspheres were diluted with 200μL of acidified Cellosolve cette (Spectrum Chemicals, Gardens, CA). The absorbance of the solution was read at 448-nm wavelength (yellow) and 670nm wavelength (blue) by a spectrophotometer (Shimadzu Columbia, MD), and the number of microspheres was calculated according to standards. Spillover between wavelengths was corrected with the matrix inversion technique. PSS was calculated as (number of microspheres) lung/(liver+lung)

***In situ* SMA perfusion**

After the BW, basal MAP, PVP and SMA flow measurement, an 18-gauge Teflon cannula was inserted in SMA to serve as the inlet. Another 16-gauge Teflon cannula as the outlet was inserted in the proximal end of the SMV vein. A ligature was tied over the proximal site of the insertion site to exclude the liver and collateral from perfusion. The animal was then transferred into the upper compartment of a warm chamber (37±0.5℃). The temperature around the perfusion area was continuously monitored with a thermometer placed inside the mesentery and maintained at approximately 37±0.5℃with a thermostatic pad and temperature-controlled infrared lamp. An open circuit perfusion was then started with Krebs solution via the mesenteric cannula by a roller pump (model 505S; Watson-Marlow Limited, Falmouth, Cornwall, UK). The perfusates was equilibrated with carbongen gas (95%O_2_-5% CO_2_) by a silastic membrane lung. The SMA cannula was opened to allow a free and complete washout of the blood. Pneumothorax was created by opening silts through the diaphragm to increase resistance in pulmonary arteries and prevent the perfusate from entering left heart chambers. The SMA was then perfused with oxygenated (95% O_2_ and 5% CO_2_) Kreb solution containing 3% wt/vol albumin (factor V bovine serum albumin; Sigma, Lt Louis, MO). The effluent of the perfused tissue was collected in a reservoir placed at the lower compartment of the warm chamber and was not recirculated. To continuously monitor and record the pressure of this territory, s Spectramed DTX transducer attached to the Gould model RS 3400 recorder (Gould Inc., Cupertino, CA) was connected to a side arm placed just proximal to the perfusion cannula, with the zero placed at the level of right atrium. After the temperature and pressure of the system were stabilized within 10 minutes, all the experiments were performed 20 minutes after starting perfusion at a constant flow rate of 15mL/min. Moreover, the perfusion flow rate was kept constant throughout the whole experiment, so the changes in perfusion flow rate was kept constant throughout the whole experiment, so the changes in perfusion pressure reflected the changes in splanchnic vascular resistance. Only one concentration-response curve was performed in each preparation. In each individual preparation, cumulative dose response curves of acetylcholine (ACh, 10^-12^ to 10^-7^M, endothelial dependent vasodilators) with phenylephrine (PEP, 10μM) pre-contraction and AVP (10^-10^ to 10^-6^ M) were measured. This concentration of PEP was found to result in 25% constrictor response in all BDL-cirrhotic SMAs. After testing experimental agents, the contracting capability of the SMA was challenged with a 125-mM potassium chloride solution at the end of experiments.

***In situ* portosystemic collateral vessels perfusion**

Briefly, both jugular veins were cannulated with 16-gauge Teflon cannulas. The abdomen was then opened and an 18-gauge Teflon cannula was inserted in the distal SMV and fixed with cyanoacrylate glue. To exclude the liver from perfusion, the second loose ligature around the portal vein was tied. After the temperature and pressure of the system were stabilized for 10 minutes, all the experiments were performed 20 minutes after starting perfusion at a constant flow rate of 12mL/min. In each individual preparation, cumulative dose response curves of acetylcholine (Ach, 10^-12^ to 10^-7^M, endothelial dependent vasodilators) with phenylephrine (PEP, 10μM) pre-contraction and AVP (10^-10^ to 10^-6^ M) were measured. This concentration of PEP was found to result in 30% constrictor response in all BDL-cirrhotic portosystemic collateral vessels. After testing experimental agents, the contracting capability of the portosystemic collateral vessels was challenged with a 125-mM potassium chloride solution at the end of experiments.

**The *in vivo* evaluation of the mesenteric vascular density with IF study**

The mesenteric angiogenesis (mesenteric window vascular length and area) were measured with flow probes and FITC-labeled CD31 IF staining as previous study [2]. Briefly, 4 to 5 vascularized mesenteric windows (wedge-shaped regions of connective tissue bordered by the intestinal wall and the ileal blood vessel pairs) were dissected free, washed in phosphate-buffered saline, dried on gelatin slides, and fixed in 100% MeOH (-20 ℃ for 30 min). Slides were then incubated overnight at 4°C with the primary antibody mouse anti-rat CD31-biotin [1:200; AbD Serotec, Oxford, UK]. Then secondary antibody [CY2-conjugated streptavidin, 1:1000; Jackson ImmunoResearch, West Grove, PA, USA] was applied for 1 hour at room temperature**.** For each mesenteric window, at least four sets of (100x)-magnification immunofluorecent images were assessed using a upright fluorescent microscope (AX80, Olympus, Japan) with charge-couple device (QICAM, High-performance IEEE 1394 FireWireTM Digital CCD Camera, Q IMAGING, BD, Canada) and threshold by Image J software (available for download from the National Institutes of Health (NIH, http://rsb.info.nih.gov/ij/). The vascular length was manually measured with the pencil tool and the vascular area automatically with histogram function, respectively. According to the information provided by QICAM, with the eyepiece 10x, the diameter of one pixel on an image taken with QICAM equals to 4.65 μm. Under 100x-magnification (objective 10x and eyepiece 10x), the diameter of one pixel would be 4.65 μm/10=0.465 μm. The vascular length could thus be determined accordingly. The unit of vascular length per unit area of mesenteric window would be μm·(μm^2^)^-1^= μm^-1^ and the vascular area per unit area of mesenteric window, actually, could be pixel·pixel^-1^ without being converted toμm^2^·(μm^2^)^-1^.

**HUVECs matrigel tube formation angiogenesis assay**

The *in vitro* matrigel tube formation angiogenesis assay kit (Kurabo, Tokyo, Japan) was used to assess the formation of capillary like structure of HUVECs. Briefly, matrigel was used to coat the wells of 24-well plates (0.25ml per well) and was left to polymerize at 37°C for 1 hour. After polymerization, the HUVEC (1×10^5^ cells/well) were incubated in growth media and allowed to attach for 24 hours. Cells were washed twice with M199 and incubated for 6 hour with M199 containing 1% fetal calf serum and antibiotics. HUVECs were pretreated with buffer, VEGF (50ng/mL), VEGF+TNFα (0.05ng/mL) and VEGF+TNFα+thalidomide (10^-3^M). After 30-hour of incubation, the HUVECs were fixed. Images were captured using an Olympus Inverted Research Microscope (Olympus, Tokyo, Japan) coupled to an Olympus C-5050 Zoom digital camera. Images were prepared in Adobe Photoshop 7.0 (Adobe, San Jose, CA, USA) and exported to an image analysis software package for identification of endothelial cell tubule-like networks. The total tubule length (≥30μm) was derived for each of the 4 randomly chosen fields, and the total area of the culture surfaces covered by HUVECs was determined in the same fields. The angiogenic index value was obtained for each culture well and compared among different pretreatment groups.

**Transwell chemotaxis filter assay for HUVECs migration**

In order to assess the acute effects of thalidomide in the TNFα-augmented VEGF-mediated HUVECs migration, thalidomide (10^-3^ M) and TNFα (0.05ng/mL) were co-administrated with VEGF (50ng/mL); and these results were compared to the vehicle-co-administrated group. HUVECs migration was assessed using a chemotaxis chamber with inserts equipped with a 8-mm-pore membrane of 0.3cm^2^ and were placed in 24-well culture dishes, forming the upper and lower compartments of the assay, respectively (Transwell, Corning Costar, Cambridge, MA, USA). The lower compartment, contained medium (buffer) alone (DMEM/0.2% BSA) or with drugs, were pre-coated with type I collagen (50 mg/L). The upper compartment of the chemotaxis filter assay are seeded with HUVECs (3-3.5×10^5^ cells/mL), which had been serum-starved for 24 hours, in 150μL of serum-free medium. The entire chamber was incubated at 37°C for another 6 hours to allow possible migration of cells. At the end of experiments, the HUVECs remaining on the upper surface of filters were removed with cotton tips. The membranes were fixed in 100% methanol, stained with May-Grunwald-Giemsa, mounted in glycergel on glass slides, and examined under microscope. HUVECs adhering to the underside of membranes were counted in 10 random high-power fields (3400) and expressed as percentages of the control (buffer only group). The mean count was determined from three independent experiments were expressed as means±SD. Then, the migration index (MI) was calculated [MI = no. of HUVECs transmigrating in the presence of buffer, VEGF, VEGF+TNFα and VEGF+TNFα+thalidomide divided by the number of HUVECs transmigrating in the absence of them (buffer only)].

**Preparation of HUVECs supernatant for various protein, *mRNA* and cytokines measurements**

Cultured HUVECs (5×10^6^) with buffer, VEGF, VEGF+TNFα and VEGF+TNFα+thalidomide pre-treatment were washed twice with ice-cold phosphate-buffered saline. Cells were lysed using RIPA buffer [20mM MOPS, 150mM NaCl, 1mM EDTA, 1% Igepal, 1% sodium deoxycholate and 0.1% SDS supplement with 1:1000 concentration of protease inhibitor mix (Sigma)]. The lysates were centrifuges at 10,400×g for 10 minutes at 4°C. The supernatant was kept at -80°C until use. Various proteins expressions were detected using the corresponding antibodies. Meanwhile, commercial available ELISA kits were also used to measure total nitric oxide (NOx), angiopoietin-1, IL-1β, and caspase-1 in supernatant of HUVECs with different treatments.

Total RNAs were obtained from cultured HUVECs with buffer, VEGF, VEGF+TNFα and VEGF+TNFα+thalidomide pre-treatments by using RNeasy extraction kit (Qiagen, Mississauga, ON, Canada). cDNA was synthesized from 1μg total RNA using the QuantiTect Reverse Transcription kit (Qiagen).

**References**

1. Abraldes JG, Iwakiri Y, [Loureiro-Silva M](http://www.ncbi.nlm.nih.gov/pubmed/?term=Loureiro-Silva%20M%5BAuthor%5D&cauthor=true&cauthor_uid=16603731), [Haq O](http://www.ncbi.nlm.nih.gov/pubmed/?term=Haq%20O%5BAuthor%5D&cauthor=true&cauthor_uid=16603731), [Sessa WC](http://www.ncbi.nlm.nih.gov/pubmed/?term=Sessa%20WC%5BAuthor%5D&cauthor=true&cauthor_uid=16603731), [Groszmann RJ](http://www.ncbi.nlm.nih.gov/pubmed/?term=Groszmann%20RJ%5BAuthor%5D&cauthor=true&cauthor_uid=16603731). Mild increases in portal pressure upregulate vascular endothelial growth factor and endothelial nitric oxide synthase in the intestinal microcirculatory bed, leading to a hyperdynamic state. ***Am J Physiol*** 2006;290:G980-987.
2. Yang M, Stapor PC, Peirce SM, Betancourt AM, Murfee WL. Rat mesentery exteriorization: a model for investigating the cellular dynamics involved in angiogenesis. ***J Vis Exp*** 2012;63:e3954.
